# A longitudinal multilevel CFA-MTMM model for interchangeable and structurally different methods

**DOI:** 10.3389/fpsyg.2014.00311

**Published:** 2014-04-17

**Authors:** Tobias Koch, Martin Schultze, Michael Eid, Christian Geiser

**Affiliations:** ^1^Department of Educational Science and Psychology, Freie Universität BerlinBerlin, Germany; ^2^Psychology Department, Utah State UniversityLogan, UT, USA

**Keywords:** multilevel structural equation modeling, longitudinal modeling, MTMM modeling, multirater data, rater bias, method effects, simulation study

## Abstract

One of the key interests in the social sciences is the investigation of change and stability of a given attribute. Although numerous models have been proposed in the past for analyzing longitudinal data including multilevel and/or latent variable modeling approaches, only few modeling approaches have been developed for studying the construct validity in longitudinal multitrait-multimethod (MTMM) measurement designs. The aim of the present study was to extend the spectrum of current longitudinal modeling approaches for MTMM analysis. Specifically, a new longitudinal multilevel CFA-MTMM model for measurement designs with structurally different and interchangeable methods (called Latent-State-Combination-Of-Methods model, LS-COM) is presented. Interchangeable methods are methods that are randomly sampled from a set of equivalent methods (e.g., multiple student ratings for teaching quality), whereas structurally different methods are methods that cannot be easily replaced by one another (e.g., teacher, self-ratings, principle ratings). Results of a simulation study indicate that the parameters and standard errors in the LS-COM model are well recovered even in conditions with only five observations per estimated model parameter. The advantages and limitations of the LS-COM model relative to other longitudinal MTMM modeling approaches are discussed.

## 1. Introduction

An increasing body of research is devoted to longitudinal data analysis examining the change and stability of a given attribute across time (see Singer and Willett, 2003; Khoo et al., 2006). The prominence of longitudinal studies may be explained by the fact that longitudinal measurement designs bear many advantages. Longitudinal measurement designs are more informative than cross-sectional studies, allowing researchers to (1) investigate change and/or variability processes, (2) test the degree of measurement invariance as well as indicator-specific effects, and (3) examine potential causal relationships (see Steyer, 1988, 2005). Over the last decades, many statistical models have been proposed for analyzing longitudinal data including multilevel as well as latent variable modeling approaches (c.f. Little et al., 2000; Singer and Willett, 2003; Rabe-Hesketh and Skrondal, 2004; Steele, 2008; Heck et al., 2013). On the other hand, only few attempts have been made to develop appropriate models for longitudinal multitrait-multimethod (MTMM) data (e.g., Kenny and Zautra, 2001; Burns and Haynes, 2006; Courvoisier et al., 2008; Grimm et al., 2009; Geiser et al., 2010; Koch, 2013).

Originally, multitrait-multimethod (MTMM) analysis was developed for scrutinizing the construct validity of social science measures (Campbell and Fiske, 1959). According to Campbell and Fiske (1959) at least two traits (e.g., empathy and aggression) and two methods (e.g., student reports and teacher reports) are required for investigating the degree of convergent and discriminant validity among different measures. Convergent validity refers to the associations (correlations) between two methods measuring the same trait (e.g., the correlation between empathy measured via student and teacher reports). Discriminant validity refers to the question of whether and to which extent methods are able to differentiate between different traits (e.g., the correlation between self-reported empathy and self-reported aggression).

Combining the advantages of longitudinal modeling approaches and MTMM modeling approaches can be fruitful. For example, longitudinal MTMM models allow researchers to investigate the construct validity of different measures across time by combining the information provided by multiple methods or reporters in a single model. This is useful because a researcher would otherwise have to estimate separate longitudinal models for each reporter and no information as to the relationship between reporters could be obtained. Moreover, longitudinal MTMM models allow modeling method effects, examining the stability and change of these method effects across time, and scrutinizing potential causes of method effects by including other (manifest of latent) variables in the model.

The purpose of the present work is to extend the range of longitudinal models for the analysis of complex longitudinal MTMM data by presenting a comprehensive modeling framework for different types of methods. Specifically, we present a new multilevel structural equation model for the analysis of longitudinal MTMM data featuring interchangeable and structurally different methods. The model is called Latent-State-Combination-Of-Methods model (LS-COM) model. The LS-COM model combines the advantages of four modeling approaches, that is, structural equation modeling, multilevel modeling, longitudinal modeling, and MTMM modeling with interchangeable and structurally different methods. In particular, the LS-COM allows researchers to (1) explicitly model measurement error, (2) specify method factors on different measurement levels, (3) analyze the convergent and discriminant validity across multiple occasions, (4) investigate change and stability of construct and methods effects across time, and (5) test important assumptions in longitudinal data analysis such as the degree of measurement invariance. The LS-COM model is formulated based on the principles of stochastic measurement theory (Zimmerman, 1975; Steyer and Eid, 2001), which has the advantage that all latent variables in the model are psychometrically well-defined as random variables with a clear meaning.

The article is structured as follows: First, we review conventional (single-method) models of longitudinal confirmatory factor analysis with a special focus on latent state (LS) models (Steyer et al., 1992). Second, we discuss current extensions of LS-modeling approaches to MTMM designs with structurally different methods. In this regard, we review the correlated state-correlated method minus one [CS-C(M-1)] model by Geiser et al. (2010). Furthermore, we explain the differences between measurement designs with structurally different methods, interchangeable methods, or a combination of both methods. We show that the CS-C(M-1) model is useful for modeling data obtained from longitudinal MTMM measurement designs with structurally different methods, but that this model is not suitable for measurement designs with a combination of structurally different and interchangeable methods. Third, we present the new LS-COM model for longitudinal MTMM designs with structurally different and interchangeable methods. The new LS-COM model fills a gap in the literature, as previous approaches to longitudinal MTMM analysis focused exclusively on structurally different methods. Fourth, we report the results of a Monte Carlo simulations study in which we examined the statistical performance of the LS-COM model. Finally, we discuss the advantages and limitations of the LS-COM model compared to other longitudinal MTMM modeling approaches.

## 2. Longitudinal confirmatory factor analysis

**Figure 1:**
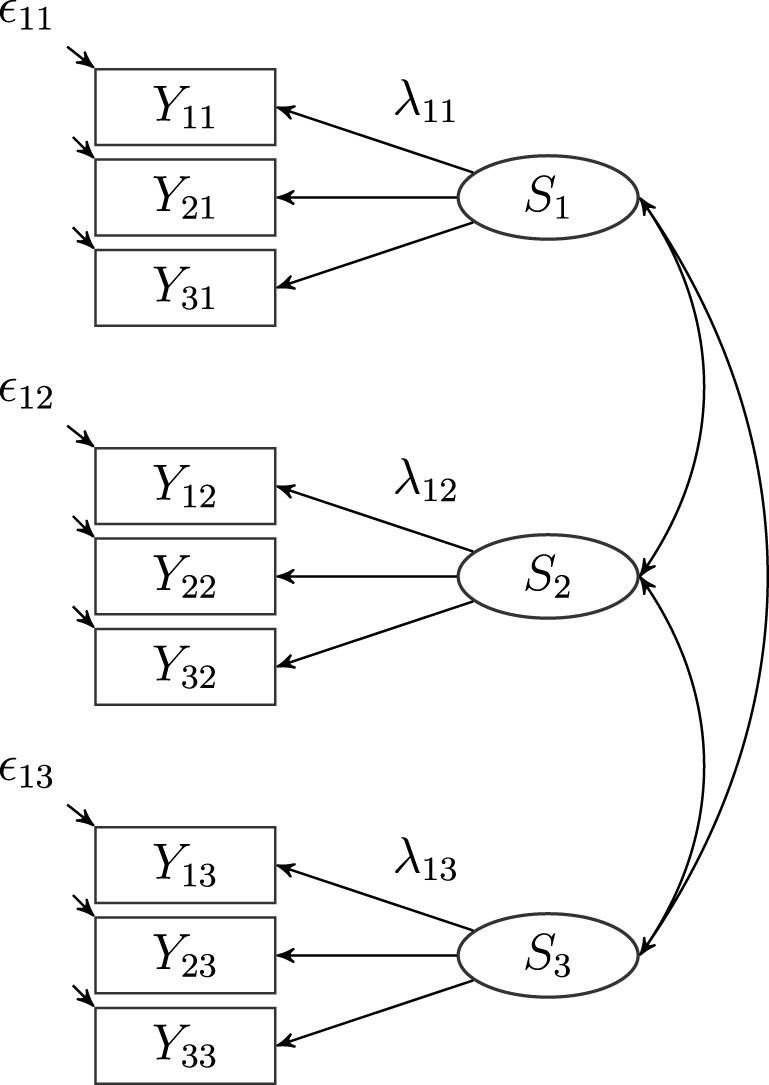
**A latent state (LS) model with three indicators (*i* = 3) and three occasions of measurement (*l* = 3), where *S*_*l*_: latent state factors, λ_*il*_: latent state factor loadings, ϵ_*il*_: measurement error variables**. Intercepts (α_*i l*_) are not depicted in the figure.

The versatility and flexibility of the CFA framework have inspired the development of different CFA models for longitudinal measurement designs, for example, autoregressive models (Hertzog and Nesselroade, 1987; Jöreskog, 1979a,b; Marsh, 1993; Eid and Hoffmann, 1998), latent state models (Steyer et al., 1992), latent change (difference score) models (Steyer et al., 1997, 2000; McArdle, 1988), latent state-trait models (Steyer et al., 1992, 1999), and latent growth curve models (McArdle and Epstein, 1987; Meredith and Tisak, 1990; Hancock et al., 2001; Bollen and Curran, 2006; Duncan et al., 2006). Most previous longitudinal models have been designed for single method measurement designs (e.g., self-reports) only. Presumably, the simplest CFA model for longitudinal data is the latent state (LS) model, which represents an extension of classical test theory to longitudinal measurement designs (see Steyer et al., 1992; Marsh, 1993; Tisak and Tisak, 2000; Geiser, 2009). The LS model is often used as a baseline model, given that it implies no restrictions with regard to the structural part of the model (see Figure 1). Hence, the LS model is often used for testing the measurement model (e.g., the validity of the assumed factor structure, measurement invariance restrictions, correlations of error variables, unidimensionality of the scales on an occasion of measurement). According to latent state theory (see Steyer et al., 1992), each observed variable *Y*_*i l*_ can be decomposed into a latent state (*S*_*i l*_, occasion-specific true score) variable and a measurement error variable ϵ_*i l*_, where *i* is the indicator (item or parcel) and *l* denotes the occasion of measurement:

(1) $$Y_{il}=S_{il}+\epsilon_{il}.$$

The latent state variable *S*_*i l*_ represents the individual state scores at a particular occasion of measurement, whereas the measurement error variables reflect unsystematic influences due to measurement error. It can be shown that the additive decomposition of the observed variables *Y*_*i l*_ into a latent state variable *S*_*i l*_ and a latent measurement error variable ϵ_*i l*_ follow directly, if both latent variables are defined in terms of conditional expectations (see Steyer, 1988, 1989; Steyer et al., 1992). In order to estimate a latent state model, it is assumed that (1) the latent state variables belonging to the same occasion of measurement are linear functions of each other (i.e., congenerity assumption):

(2) $$S_{il}=\alpha_{ii^{\prime }l}+\lambda_{ii^{\prime }l}S_{i^{\prime }l},$$

and that (2) the measurement error variables [i.e., *C*ov(ϵ_*i l*_, ϵ_*i l*_) for (*i*, *l*) ≠ (*i*′, *l*′)] are uncorrelated with each other. Equation (2) states that the latent state variables are linear functions of each other and only differ by an additive constant α_*ii′l*_ and multiplicative constant λ_*ii′l*_. With respect to this assumption, it is possible to show that Equation (2) is equivalent to *S*_*i l*_ = α_*i l*_ + λ_*i l*_*S*_*l*_. Hence, the general measurement equation of a latent state model with common latent state factors can be written as follows:

(3) $$Y_{il}=\alpha_{il}+\lambda_{il}S_{l}+\epsilon_{il}.$$

α_*i l*_ is the intercept and λ_*i l*_ is the factor loading parameter pertaining to the latent state factors. As a consequence of the assumptions explained above, the total variance of the observed variables can be decomposed as follows:

(4) $$Var(Y_{il})=\lambda_{il}^{2}Var(S_{l})+Var(\epsilon_{il}).$$

The reliability of each observed variable is then given by:

(5) $$Rel(Y_{il})=\frac{\lambda_{il}^{2}Var(S_{l})}{Var(Y_{il})}.$$

Figure 1 shows a path diagram of the latent state model for three indicators and three occasions.

The correlations between the latent state factors *S*_*l*_ characterize the stability of interindividual differences on the given attribute (see Figure 1). High correlations reflect that individual differences with regard to a particular attribute (construct) are rather stable over time. Researchers may also investigate mean change of a given construct across time. For meaningful interpretations of latent mean change, we recommend that measurement invariance (MI) should be tested and that researchers should at least establish strong MI (e.g., Meredith, 1993; Widaman and Reise, 1997; Millsap, 2012).

Strong MI can be established by imposing the following restrictions:

1. The intercepts of the observed variables α_*i l*_ have to be set equal across time (i.e., α_*i l*_ = α_*i l*′_ = α_*i*_).
2. The factor loading parameters λ_*i l*_ have to be set equal across time (i.e., λ_*i l*_ = λ_*i l*′_ = λ_*i*_) and one factor loading parameter on each occasion of measurement has to be fixed to the same value (e.g., λ_1_ = 1).
3. The mean of the first latent state factor has to set to be zero [i.e., *E*(*S*_1_) = 0].
4. The mean of the remaining latent state factors can be freely estimated [i.e., *E*(*S*_*l*_) ≠ 0].

Strong MI is a prerequisite for studying true mean change (Steyer et al., 1997, 2000)^1^. Restrictions 3 and 4 allow examining mean change relative to the first measurement occasion^2^. Although the LS model (as well as other longitudinal CFA models) offers many advantages such as analyzing change and stability of an attribute apart from measurement error influences and testing the degree of measurement invariance, the LS model is limited in terms of incorporating data from multiple raters or methods, because the model does not contain method factors. In order to study the convergent and discriminant validity in longitudinal MTMM designs, more sophisticated models are needed.

## 3. Longitudinal CFA-MTMM models

According to Eid and Diener (2006) multimethod measurement designs overcome many limitations of single method measurement designs and should therefore be preferred whenever possible. With respect to longitudinal CFA-MTMM models it is possible to (1) investigate the convergent and discriminant validity at each occasion of measurement and across different occasions of measurement, (2) study change and stability of construct and method effects across time, (3) model measurement error, (4) investigate the generalizability of method effects, and (5) test important assumptions such as measurement invariance and/or indicator-specific effects.

Today, MTMM measurement designs are commonly analyzed using confirmatory factor analysis (CFA-MTMM models) with multiple indicators in each trait-method unit (e.g., Widaman, 1985; Marsh and Hocevar, 1988; Marsh, 1989; Wothke, 1995; Dumenci, 2000; Eid, 2000; Eid et al., 2003, 2006). Up to now, only few CFA-MTMM models have been proposed for the analysis of longitudinal data (e.g., Kenny and Zautra, 2001; Burns and Haynes, 2006; Courvoisier et al., 2008; Grimm et al., 2009; Geiser et al., 2010; Koch, 2013).

One exception is the study by Grimm et al. (2009) who recently proposed a longitudinal CFA-MTMM model combining the correlated trait-correlated method (CT-CM) approach (Widaman, 1985; Marsh and Grayson, 1995) and the latent growth curve modeling approach (e.g., McArdle and Epstein, 1987; Meredith and Tisak, 1990). However, results of previous studies have shown that the CT-CM modeling approach is associated with various theoretical and empirical problems (e.g., Marsh, 1989; Kenny and Kashy, 1992; Marsh and Grayson, 1994; Steyer, 1995; Eid, 2000; Geiser et al., in press). In addition, the CFA-MTMM model by Grimm et al. (2009) is limited to single-indicator measurement designs and does not allow specifying trait-specific method factors.

**Figure 2:**
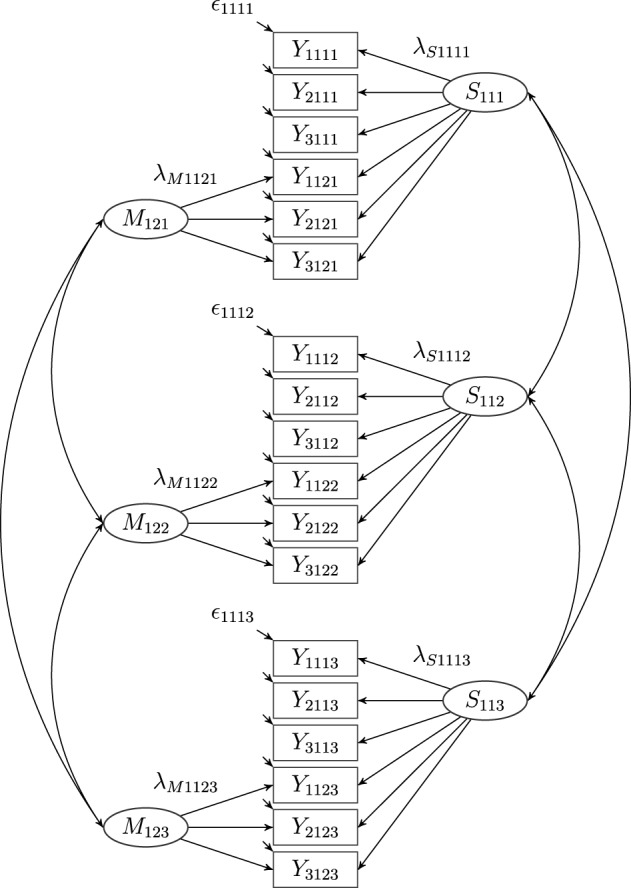
**A latent correlated state correlated method minus one [CS-(*M*-1)] model with three indicators (*i* = 3), one construct (*j* = 1), two methods (*k* = 2) and three occasions of measurement (*l* = 3), where *Y*_*ijkl*_: observed variables, *S*_*jkl*_: latent state factors, λ_*Sijkl*_: state factor loadings, *M*_*jkl*_: latent method factors, λ_*Mijkl*_: method factor loadings, ϵ_*ijkl*_: measurement error variables**. Intercepts (α_*ijkl*_) are not depicted in the figure.

Geiser et al. (2010) developed a longitudinal CFA-MTMM model [called correlated state-correlated method minus one, CS-C(M-1) model] that combines LS theory with the correlated trait-correlated method minus one [CT-C(M-1)] approach (Eid, 2000; Eid et al., 2003, see Figure 2). In this model, one method has to be chosen as reference method which all other methods are compared to. The common latent state factor is the state factor of the reference method. Each observed variable of a non-reference method is decomposed into three parts: (1) a part that is predictable by the common state factor, (2) a part that is method-specific, and (3) measurement error. One advantage of the CS-C(M-1) model is that all latent variables are well-defined with a clear and unambiguous interpretation (Geiser, 2009). The CS-C(M-1) model also overcomes many limitations of previous CFA-MTMM modeling approaches. For example, the CS-C(M-1) model allows specifying trait-specific method factors using multiple indicators per trait-method unit (TMU) and separating the observed variance into trait, method, and measurement error variance. According to the results of simulation studies (Crayen, 2008; Geiser, 2009), the CS-C(M-1) model performs well in many conditions.

However, the CS-C(M-1) model cannot be applied to all possible longitudinal MTMM measurement designs. In particular, the CS-C(M-1) model is not suitable for MTMM measurement designs combining structurally different and interchangeable methods. In the next section, the differences between measurement designs with structurally different and interchangeable methods are explained in greater detail.

## 4. Different types of methods

**Figure 3:**
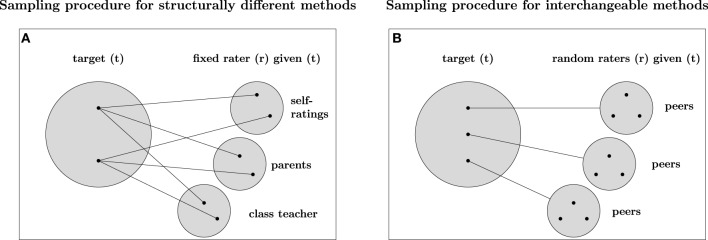
**Sampling procedure for different types of methods**. Panel **(A)** refers to the sampling procedure of measurement designs with structurally different methods. Panel **B** refers to the sampling procedure of measurement designs with interchangeable methods. The big gray filled circles are the sets of possible targets, the small gray filled circles are the sets of possible methods (raters). The black filled circles represent possible observation units belonging to the set of targets or raters. Structurally different (fixed) methods are indicated by straight lines connecting raters and targets directly. Interchangeable (random) methods are illustrated by straight lines connecting a target with a particular set of possible raters by a black line.

Eid et al. (2008) clarified that the type of method used in a study is of particular importance for defining appropriate CFA-MTMM models. More specifically, Eid et al. (2008) showed that measurement designs with (a) interchangeable methods, (b) structurally different methods, and (c) a combination of structurally different and interchangeable methods imply different sampling procedures and therefore require different CFA-MTMM models. According to Eid et al. (2008), interchangeable methods are methods that can be randomly sampled from a set of similar methods. Consider, for example, multiple peer ratings of students′ empathy. Both, peer ratings and subordinates′ ratings can be considered as interchangeable, because they have more or less the same access to the target′s behavior (Eid et al., 2008). Figure 3B illustrates the sampling procedure for interchangeable methods. According to Figure 3B, measurement designs with interchangeable methods imply a multistage sampling procedure (Eid et al., 2008; Koch et al., in press). First, a target (*t*, e.g., teacher) is randomly chosen from a set of all possible targets (*t* ∈ *T*, i.e., all teachers). Second, multiple (e.g., three) students (e.g., Edgar, Emily, and Mark) are randomly sampled from the same target-specific rater set *R*_*t*_. Therefore, measurement designs with interchangeable methods imply a multilevel data structure (Eid et al., 2008).

In contrast, measurement designs with structurally different methods (see Figure 3A) use methods that are not randomly sampled out of a common set of similar methods (raters). For example, structurally different methods such as self-ratings, parent ratings, and the ratings of the class teacher do not stem from the same group of methods, but differ in many ways. As a consequence, measurement designs with structurally different methods can usually be modeled with single-level factor models [e.g., CS-(M-1) model]. An increasing number of studies use a combination of structurally different and interchangeable methods. For example, in organizational psychology it is very common to use self-reports, supervisor reports, and interchangeable colleague reports (so-called 360° feedback designs). In educational and developmental psychology, many researchers use student reports, teacher and parent reports, as well as interchangeable peer reports. All of these designs imply a combination of structurally different and interchangeable methods.

## 5. The need for longitudinal multilevel CFA-MTMM models

So far, no appropriate CFA model has been proposed for longitudinal MTMM data combining structurally different and interchangeable methods. Researchers who use such MTMM measurement designs (e.g., longitudinal multisource feedback designs with different types of raters) are therefore forced to either aggregate the interchangeable ratings into a single score or analyze both types of methods (structurally different and interchangeable methods) in separate models. The aggregation of level one units (here interchangeable methods) has been associated with various methodological shortcomings, such as, interpretation problems (e.g., ecological fallacy), loss of information, smaller sample size, larger standard errors, and loss of power (Hox, 2010; Snijders and Bosker, 2011). If both types of methods are analyzed separately, then researchers are not able to integrate (or compare) the information of both types of methods (rater groups) in the same model. For example, convergent validity of interchangeable peer reports and self-reports could be examined. Given that many researchers increasingly apply measurement designs with a combination of structurally different and interchangeable methods, there is a need for developing new methods for the analysis of such complex MTMM measurement designs. In the next section, we present the LS-COM model, which integrates LS theory and the CS-C(M-1) modeling approach for a combination of different types of methods. In addition, we present the results of a Monte Carlo simulation study, in which we examined the statistical performance of the LS-COM model under different conditions.

## 6. The latent state combination of methods (LS-COM) model

The LS-COM allows researchers

1. to scrutinize the degree of measurement invariance across time,
2. to test mean changes of particular constructs,
3. to examine the stability and change of construct and method effects across time,
4. to investigate the psychometric properties (e.g., the convergent and discriminant validity and reliability) of the given measures on each occasion of measurement and across occasions of measurement, and
5. to scrutinize the generalizability of method effects across different methods and/or different constructs.

Similar to the CS-C(M-1) model, we define the LS-COM model in different steps.

### 6.1. Step 1: choice of reference method and basic decomposition

In the first step, a reference or gold-standard method has to be chosen. The remaining methods serve as non-reference methods. The reference method is often a method that is either seen as most valid by the researcher based on theory or prior empirical results or a method that is particularly outstanding or special relative to the other methods (e.g., objective IQ tests versus self-ratings of intelligence). In the LS-COM model either one of the structurally different or the set of interchangeable methods can be chosen as reference method. For the sake of simplicity, we define the LS-COM model for two structurally different methods (method 1 = self-report, method 2 = parent report) and one set of interchangeable methods (method 3 = multiple peer reports for a student). Note that the LS-COM model is not restricted in terms of the number of structurally different methods. Moreover, we chose the first method (a structurally different method, e.g., self-reports) as reference method and assume that there is only one parent report for each target. Pham et al. (2012) as well as Schultze (2012) show how the set of interchangeable methods can be chosen as reference method. The observed variables of each method can be decomposed into a latent state and a latent measurement error variable:

(6) $$\text{Level }2:\;Y_{tij1l}=S_{tij1l}+\epsilon_{tij1l},\;(\text{structurally different method }1)$$

(7) $$\text{Level }2:\;Y_{tij2l}=S_{tij2l}+\epsilon_{tij2l},\;(\text{structurally different method }2)$$

(8) $$\text{Level }1:\;Y_{rtij3l}=S_{rtij3l}+\epsilon_{rtij3l}.\;(\text{set of interchangeable methods})$$

The index *i* represents the indicators, *j* is the construct, *k* is the method, and *l* is the occasion of measurement. In addition, the indices *r* for rater and *t* for target are required. The reason is that the interchangeable raters *r* are nested within different targets *t*. Hence, the observed variables of the self-reports and parent reports are measured on Level 2 (the target level), whereas the observed variables pertaining to the interchangeable methods (peer reports) are measured on Level 1 (the rater level). A value of the target-specific latent state variables *S*_*tijkl*_ is the true score of target *t* with respect to indicator *i*, construct *j*, method *k* (i.e., self-report or parent report), and occasion of measurement *l*. The rater-specific latent state variables *S*_*rtijkl*_ reflect the (method-specific) true peer rating of a rater *r* for a particular target *t* on indicator *i*, construct *j*, and occasion of measurement *l*. The measurement error variables on both levels are represented by ϵ_*tijkl*_ (Level 2) and ϵ_*rtijkl*_ (Level 1). In the Appendix A in Supplementary Material, we show how the latent state and measurement error variables are formally defined in terms of conditional expectations.

### 6.2. Step 2: definition of rater-specific latent method variables on level 1

In the second step, rater-specific (Level 1) latent method variables are defined for the interchangeable methods (i.e., multiple peer reports). This is possible given that multiple peers *r* rate each target *t* on different items (indicators: *i*). Therefore, the rater-specific latent state variables can be decomposed into a rater-unspecific latent state *S*_*tij*3*l*_ variable and a rater-specific method *UM*_*rtij*3*l*_ variable.

(9) $$Y_{rtij3l}=S_{rtij3l}+\epsilon_{rtij3l}$$

(10) $$S_{rtij3l}=S_{tij3l}+UM_{rtij3l}$$

(11) $$Y_{rtij3l}=S_{tij3l}+UM_{rtij3l}+\epsilon_{rtij3l}.$$

A value of the latent state variables *S*_*tij*3*l*_ can be conceived as the expected peer rating of the target *t* across the true occasion-specific peer ratings for that target. That is, the latent state variables *S*_*tij*3*l*_ can be conceived as the average peer rating and are thus variables on Level 2. A value of the latent unique method variables *UM*_*rtij*3*l*_ is the true occasion-specific deviation of a particular rater from this true mean. Hence, a value of the *UM*_*rtij*3*l*_-variables represents the over- or underestimation of the true expected peer rating by a particular rater *r*. Positive values indicate an overestimation, whereas negative values indicate an underestimation of the true expected peer rating by a particular rater. Given that the unique method *UM*_*rtij*3*l*_-variables are defined as latent residual variables, the general properties of residual variables hold. That means that the unique method *UM*_*rtij*3*l*_-variables are uncorrelated with the Level 2 latent state *S*_*tij*3*l*_ variables [i.e., *Cor*(*S*_*tij*1*l*_, *UM*_*rtj*3*l*_) = 0] and have an expectation (mean) of zero [i.e., *E*(*UM*_*rtj*3*l*_) = 0]. Moreover, as in classical multilevel (structural equation) models, it is assumed that the Level 1 residuals (here: the *UM*_*rtij*3*l*_-variables) are independently and identically distributed on Level 1 (i.e., iid-assumption).

### 6.3. Step 3: latent regressions and definition of latent method variables on level 2

Given that all latent state variables *S*_*tijkl*_ are now measured on the same level (Level 2; the target level), it is possible to contrast the latent state variables pertaining to different types of methods against each other. Following the original CT-C(M-1) approach for structurally different methods (Eid, 2000; Eid et al., 2003, 2008), the latent state variables pertaining to the non-reference methods are regressed on the latent state variables pertaining to the reference method (in this example self-reports):

(12) $$E(S_{tij2l}|S_{tij1l})=\alpha_{ij2l}+\lambda_{Sij2l}S_{tij1l},\;(\text{parent reports})$$

(13) $$E(S_{tij3l}|S_{tij1l})=\alpha_{ij3l}+\lambda_{Sij3l}S_{tij1l}.\;(\text{peer reports})$$

The (independent) latent state variable *S*_*tij*1*l*_ in the latent regression analysis denotes the occasion-specific true score measured by the reference method (e.g., self-reports). The residuals of the latent regression analyses are defined as latent method variables. These method variables are also measured on the target level (Level 2). With regard to the structurally different non-reference method (e.g., parent reports), the method variables can be defined as follows:

(14) $$M_{tij2l}\equiv S_{tij2l}-E(S_{tij2l}|S_{tij1l})=S_{tij2l}-(\alpha_{ij2l}+\lambda_{Sij2l}S_{tij1l}).$$

The method variables *M*_*tij*2*l*_ represent that part of the true parent reports that cannot be predicted by the self-reports. In other words, these method variables capture the occasion-specific part of the parent report that cannot be predicted by the self-report. As consequence of the definition of the *M*_*tij*2*l*_-variables as residual variables the latent method variables are uncorrelated with the latent state variables [i.e., *Cor*(*S*_*tij*1*l*_, *M*_*tj*2*l*_) = 0] and have an expectation (mean) of zero [i.e., *E*(*M*_*tj*2*l*_) = 0]. For the set of interchangeable methods (e.g., peer reports), the method variables can be defined as follows:

(15) $$CM_{tij3l}\equiv S_{tij3l}-E(S_{tij3l}|S_{tij1l})=S_{tij3l}-(\alpha_{ij3l}+\lambda_{Sij3l}S_{tij1l}).$$

The method variables *CM*_*tij*3*l*_ represent that part of the true expected peer ratings that is not shared with self-report on the same occasion of measurement. The common method variable is called common method variable, given that they represent the perspective of the peers that is shared by all peers, but is not shared with the self-reports on a particular occasion of measurement. By definition the latent common method variables are uncorrelated with the corresponding latent state variables of the reference method [i.e., *Cor*(*S*_*tij*1*l*_, *CM*_*tj*3*l*_) = 0] and have an expectation (mean) of zero [i.e., *E*(*CM*_*tj*3*l*_) = 0]. Moreover, the following correlations are assumed to be zero in the LS-COM model:

(16) $$Cor(S_{tij1l},UM_{rtj^{\prime }3l^{\prime }})=0,$$

(17) $$Cor(CM_{tij3l},UM_{rtj^{\prime }3l^{\prime }})=0,$$

(18) $$Cor(M_{tij2l},UM_{rtj^{\prime }3l^{\prime }})=0,$$

(19) $$Cor(\epsilon_{rt(ijkl)},\epsilon_{rt{\left(ijkl\right)}^{\prime }})=0,$$

(20) $$Cor(\epsilon_{t(ijkl)},\epsilon_{t{\left(ijkl\right)}^{\prime }})=0,$$

(21) $$Cor(\epsilon_{rt(ijkl)},\epsilon_{rt(i^{\prime }j^{\prime }k^{\prime }l^{\prime })})=0.$$

According to Equations (16–18), it is assumed that the Level 1 unique method variables are uncorrelated with all variables on Level 2 (i.e., latent state, latent common method, and latent method variables). Equations (19–21) imply that all measurement error variables belonging to different indicators, different constructs, different methods, and different occasions of measurement are uncorrelated with each other.

### 6.4. Step 4: definition of latent method factors

In order to define latent method factors, it is assumed that the latent method variables of the same method only differ by multiplicative constants (i.e., λ_*Mij*2*l*_, λ_*CMij*3*l*_, λ_*UMij*3*l*_). According to these assumptions, it is possible to define common latent method factors that are homogeneous across different indicators (i.e., *M*_*tj*2*l*_, *CM*_*tj*3*l*_, *UM*_*rtj*3*l*_):

(22) $$\text{Level }2:\;M_{tij2l}=\lambda_{Mij2l}M_{tj2l},$$

(23) $$\text{Level }2:\;CM_{tij3l}=\lambda_{CMij3l}CM_{tj3l},$$

(24) $$\text{Level }1:UM_{rtij3l}=\lambda_{UMij3l}UM_{rtj3l}.$$

The above Equations (22–24) state that the method effects are now measured by latent method factors that are common to all indicators.

### 6.5. Step 5: definition of latent state factors

Following a similar logic, it is possible to construe a latent state factor *S*_*tj*1*l*_ that is common to all indicators:

(25) $$S_{tij1l}=\alpha_{ij1l}+\lambda_{Sij1l}S_{tj1l}.$$

Overall, the general measurement equation of the LS-COM model for three methods (e.g., *k* = 1 = self-report, *k* = 2 = parent report, *k* = 3 = peer reports) and latent state factors (*S*_*tj*1*l*_) can be expressed by:

(26) $$Y_{tij1l}=\alpha_{ij1l}+\lambda_{Sij1l}S_{tj1l}+\epsilon_{tij1l},$$

(27) $$Y_{tij2l}=\alpha_{ij2l}+\lambda_{Sij2l}S_{tj1l}+\lambda_{Mij2l}M_{tj2l}+\epsilon_{tij2l},$$

(28) $$Y_{rtij3l}=\alpha_{ij3l}+\lambda_{Sij3l}S_{tj1l}+\lambda_{CMij3l}CM_{tj3l}+\lambda_{UMij3l}UM_{rtj3l}+\epsilon_{rtij3l}.$$

Equation (26) states that the reference method (e.g., self-report) indicators are only measured by a latent reference state factor *S*_*tj*1*l*_ with an intercept α_*ij*1*l*_ and factor loading parameter λ_*Sij*1*l*_ and a latent measurement error variable ϵ_*tij*1*l*_. According to Equation (27) the indicators pertaining to a structurally different non-reference method (e.g., parent reports) are measured by the latent reference state factor *S*_*tj*1*l*_ (with an intercept α_*ij*2*l*_ and factor loading parameter λ_*Sij*2*l*_), a latent method factor *M*_*tj*2*l*_ (with a factor loading parameter λ_*Mij*2*l*_), and a measurement error variable ϵ_*tij*2*l*_. Finally, Equation (28) states that the indicators belonging to the interchangeable non-reference method (e.g., peer reports) are measured by the latent reference state factor *S*_*tj*1*l*_ (with a corresponding intercept α_*ij*3*l*_ and factor loading parameter λ_*Sij*3*l*_*S*_*tj*1*l*_), a latent common method factor *CM*_*tj*3*l*_ at Level 2 and a latent unique method factor *UM*_*rtj*3*l*_ at Level 1 (with corresponding factor loading parameters λ_*CMij*3*l*_ and λ_*UMij*3*l*_), as well as a measurement error variable ϵ_*rtij*3*l*_.

## 7. Variance decomposition

Based on the definition of the LS-COM model each indicator′s variance can be decomposed as follows:

(29) $$Var(Y_{tij1l})=\lambda_{Sij1l}^{2}Var(S_{tj1l})+Var(\epsilon_{tij1l}),$$

(30) $$Var(Y_{tij2l})=\lambda_{Sij2l}^{2}Var(S_{tj1l})+\lambda_{Mij2l}^{2}Var(M_{tj2l})+Var(\epsilon_{tij2l}),$$

(31) $$\begin{array}{l} Var(Y_{rtij3l})=\lambda_{Sij3l}^{2}Var(S_{tj1l})+\lambda_{CMij3l}^{2}Var(CM_{tj3l})\\ \;+\lambda_{UMij3l}^{2}Var(UM_{rtj3l})+Var(\epsilon_{rtij3l}). \end{array}$$

Based on the above variance decomposition (see Equations 29–31), it is possible to define different coefficients for quantifying convergent validity, method-specificity and reliability (see Table 1). In contrast to the CS-C(M-1) model, the LS-COM model allows calculating Level 2 and Level 1 variance coefficients, because it contains method factors at both Level 1 (*UM*_*rtj*3*l*_) and Level 2 (*CM*_*tj*3*l*_).

**Table 1:** **Variance components of the non-reference method indicators in LS-COM model**.

**Level**	**Method**	**Definition**
**CONSISTENCY**		
Level 2	Struct. different	$Con(S_{tij2l})=\frac{\lambda_{Sij2l}^{2}Var(S_{tij1l})}{Var(Y_{tij2l})-Var(\epsilon_{tij2l})}$
Level 2	Interchangeable	$Con(S_{tij3l})=\frac{\lambda_{Sij3l}^{2}Var(S_{tij1l})}{\lambda_{Sij3l}^{2}Var(S_{tij1l})+\lambda_{CMij3l}^{2}Var(CM_{tj3l})}$
Level 1	Interchangeable	$Con(S_{rtij3l})=\frac{\lambda_{Sij3l}^{2}Var(S_{tij1l})}{Var(Y_{rtij3l})-Var(\epsilon_{rtij3l})}$
Level 1	Interchangeable	$RC(S_{rtij3l})=\frac{\lambda_{Sij3l}^{2}Var(S_{tij1l})+\lambda_{CMij3l}^{2}Var(CM_{tj3l})}{Var(Y_{rtij3l})-Var(\epsilon_{rtij3l})}$
**METHOD SPECIFICITY**		
Level 2	Struct. different	$MS(S_{tij2l})=\frac{\lambda_{Mij2l}^{2}Var(M_{tj2l})}{Var(Y_{tij2l})-Var(\epsilon_{tij2l})}$
Level 2	Interchangeable	$CMS(S_{rtij3l})=\frac{\lambda_{CMij3l}^{2}Var(CM_{tj3l})}{Var(Y_{rtij3l})-Var(\epsilon_{rtij3l})}$
Level 1	Interchangeable	$UMS(S_{rtij3l})=\frac{\lambda_{UMij3l}^{2}Var(UM_{rtj3l})}{Var(Y_{rtij3l})-Var(\epsilon_{rtij3l})}$
**RELIABILITY**		
Level 2	Struct. different	$Rel(Y_{tij2l})=1-\frac{Var(\epsilon_{tij2l})}{Var(Y_{tij2l})}$
Level 1	Interchangeable	$Rel(Y_{rtij3l})=1-\frac{Var(\epsilon_{rtij3l})}{Var(Y_{rtij3l})}$

In total, four different consistency coefficients [i.e., *Con*(*S*_*tij*2*l*_), *Con*(*S*_*tij*3*l*_), *Con*(*S*_*rtij*3*l*_), and the rater consistency coefficient *RC*(*S*_*rtij*3*l*_)] can be defined. The Level 2 consistency coefficient *Con*(*S*_*tij*2*l*_) for the indicators pertaining to the structurally different non-reference methods represents the amount of true interindividual differences of the non-reference method (e.g., parent report) that can be explained by the reference method (self-report). The Level 1 consistency coefficient *Con*(*S*_*rtij*3*l*_) for the indicators pertaining to the interchangeable non-reference methods (e.g., peer reports) reflects the amount of true interindividual differences of the individual peer reports that can be explained by the reference method (here: self-report).

Sometimes researchers rather seek to know whether peers in general agree with the student self-reports. In such cases, they may calculate the Level 2 consistency coefficient *Con*(*S*_*tij*3*l*_) for the indicators pertaining to the set of interchangeable methods. This consistency coefficient captures the amount of true interindividual differences of the expected peer ratings (the entire peer-group) that can be explained by the reference method (here: self-reports). Moreover, the true rater consistency coefficient *RC*(*S*_*rtij*3*l*_) is defined as the proportion of true interindividual differences of the peer ratings that are free of measurement error and rater-specific effects. The rater consistency coefficient indicates how much true variance of a non-reference indicator is due to the overall amount of rater agreement (peers and self-ratings) and not due to measurement error influences or individual (rater-specific) influences. The true rater consistency coefficient can also be interpreted as true intra-class correlation. Moreover, three different method-specificity coefficients [i.e., *MS*(*S*_*tij*2*l*_), *CMS*(*S*_*rtij*3*l*_), and *UMS*(*S*_*rtij*3*l*_)] can be analyzed. The method specificity coefficients *MS*(*S*_*tij*2*l*_) indicate the degree or true variance of a non-reference method indicator pertaining to a structurally different method (e.g., parent reports) that is not determined by the reference method (e.g., self-report). The unique method specificity coefficient *UMS*(*S*_*rtij*3*l*_) represents the proportion of true variance of a non-reference method indicator pertaining to the interchangeable methods that is neither shared with the self-reports nor with other peers. Hence, this coefficient reflects the unique view of a particular rater on a particular occasion of measurement. The common method specificity coefficient *CMS*(*S*_*rtij*3*l*_) reflects the proportion of true interindividual differences of the peer ratings that cannot be explained the reference method (here: self-reports), but that is shared by other peers (Eid et al., 2008). Hence, this coefficient can also be interpreted as “rater consensus” with respect to the peer ratings that is not shared with the reference method.

## 8. Permissible correlations

**Figure 4:**
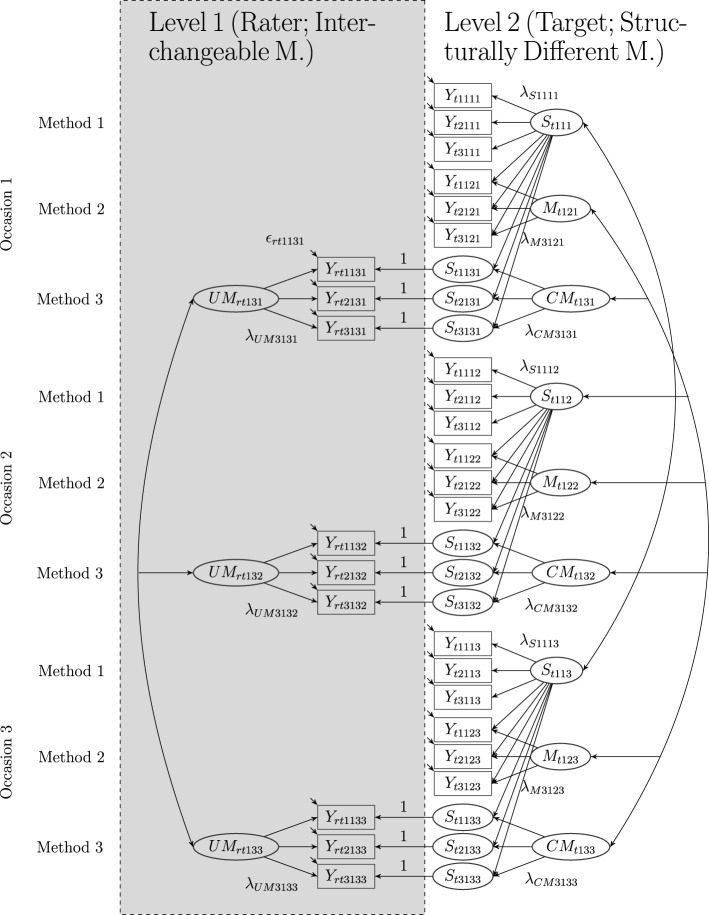
**The LS-COM model with latent state factors and three indicators (*i* = 3), one construct (*j* = 1), two structurally different and one set of interchangeable methods (*k* = 3), and three occasions of measurement (*l* = 3), where *r*: rater and *t*: target**. *Y*_*rtijkl*_: observed variables at Level 1, *Y*_*tijkl*_: observed variables at Level 2, *S*_*tjkl*_: latent state factors, λ_*Sijkl*_: state factor loadings,*M*_*tjkl*_: latent method factors, λ_*Mijkl*_: method factor loadings, *CM*_*tjkl*_: latent common method factors, λ_*CMijkl*_: common method factor loadings, *UM*_*rtjkl*_: latent unique method factors, λ_*UMijkl*_: unique method factor loadings, ϵ_*rtijkl*_: measurement errors at Level 1, and ϵ_*tijkl*_: measurement errors at Level 2. Intercepts (α_*ijkl*_) are not depicted in the figure. In this example, one of the structurally different methods (Method 1) serves as reference method.

Figure 4 shows a path diagram of a LS-COM model with three indicators per TMU, one construct, three methods and three occasions of measurement. As illustrated in the figure, the latent state factors can be correlated with each other (see Figure 4). Correlations between latent state factors pertaining to the same construct (e.g., empathy) and different occasions of measurement can be interpreted as indicators of construct stability. High positive correlations indicate that the construct is rather stable across time. Correlations between latent state factors pertaining to different constructs and the same occasion of measurement can be interpreted in terms of discriminant validity. High correlations indicate low discriminant validity at a given moment in time. Correlations between latent state factors pertaining to different constructs and different measurement occasions may be interpreted as coefficients of predictive validity. For example, students′ self-reported level of empathy measured on the first occasion of measurement (*S*_*t*111_) may be indicative for the self-reported level of relational aggression measured on the second occasion of measurement (*S*_*t*212_). Moreover, correlations between the occasion-specific latent method factors pertaining to the same measurement level are permitted in the LS-COM model.

The stability of method (rater) effects can be investigated by correlations between method factors pertaining to the same construct, same method, and different occasions of measurement. For example, correlations between the unique method factors *UM*_*rtj*3*l*_ and *UM*_*rtj*3*l*′_ (where *l* ≠ *l*′) indicate to what extent the individual rater-specific effects remain stable across time. Following a similar logic, the correlations between common method factors *CM*_*tj*3*l*_ and *CM*_*tj*3*l*′_ (where *l* ≠ *l*′) indicate to what extent the common rater effects (i.e., rater effects that are not shared with the self-report, but are shared with all other raters belonging to a particular target) remain stable across time. The generalization of method effects across constructs is indicated by correlations between method factors pertaining to different constructs (e.g., empathy and relational aggression). For example, a negative correlation between the method factors pertaining to the peer reports assessing empathy and relational aggression would indicate that peers who tend to underestimate students′ self-reported empathy level, tend to overrate students′ self-reported aggression level and vice versa. The generalization of method effects across different methods (rater types) is indicated by the correlation between method factors pertaining to different methods (e.g., parents and peers). For example, correlations between the two method factors *M*_*tj*2*l*_ and *CM*_*tj*3*l*_ indicate whether peers and parents deviate in a similar ways (how a shared bias) from the self-reports on occasion of measurement *l*.

### 8.1. Mean change

In addition to the investigation of the latent correlation as well as the variance components (provided in Table 1), many researchers seek to scrutinize the mean change of a particular construct across time. According to Equations (26–28), the expectation (mean) of the latent state factor can be identified as follows:

(32) $$E(Y_{tij1l})=E(\alpha_{ij1l})+E(\lambda_{tij1l}S_{tj1l})+E(\epsilon_{tij1l}),$$

(33) $$\;=\alpha_{ij1l}+\lambda_{tij1l}E(S_{tj1l}).$$

Given that ϵ_*tij*1*l*_ is a zero-mean normally distributed residual variable, the latent mean of the latent state variables can be identified by setting one intercept for each latent state factor to zero [e.g., *E*(α_1*j*1*l*_) = 0] and the corresponding factor loading to one [i.e., λ_*S*1*j*1*l*_ = 1]. Another possibility is to set the intercepts and factor loadings equal across time (i.e., assuming strong measurement invariance; see below) and to set the latent mean of the first latent state factor to zero. Then, the latent means of the remaining latent state variables reflect the true mean change of construct *j* from occasion of measurement 1 to occasion of measurement *l*.

### 8.2. Testing measurement invariance across time

Measurement invariance (MI) plays an important role in longitudinal analysis (Meredith, 1993; Widaman and Reise, 1997; Geiser et al., 2014). According to Widaman and Reise (1997) four different degrees of MI can be distinguished: (1) configural MI, (2) weak MI, (3) strong MI, and (4) strict MI. Configural MI implies that the number of factors as well as the factor structure as such is similar across different measurement occasions. In addition to configural MI, weak MI requires that the factor loading parameters for each indicator *i* are equal across different occasions of measurement. In addition to weak MI, strong MI assumes that the intercepts of indicators *i* are equal across different occasions of measurement. The most restrictive form of longitudinal MI (i.e., strict MI) implies that, in addition to the previous restrictions, the residual variances of indicators *i* are also equal across time. In the current work, we focus on one (not all possible) MI restriction that can be specified and empirically tested. In particular, we discuss the minimal set of restrictions that are necessary to meaningfully study mean change (i.e., strong MI) with respect to the reference method (e.g., self-reports). To meaningfully interpret mean change in the reference state factors, we recommend that the following MI restrictions be tested:

(34) $$\alpha_{ij1l}=\alpha_{ij1l^{\prime }}=\alpha_{ij1},\text{ where}\;l\neq l^{\prime },$$

(35) $$\lambda_{Sij1l}=\lambda_{Sij1l^{\prime }}=\lambda_{Sij1},\text{ where}\;l\neq l^{\prime }.$$

The above restrictions (34) and (35) state that the intercept and factor loading parameters of the reference state factors are time-invariant. These assumptions imply that the scale on which the reference latent state factors are measured does not change across time. Hence, researchers who are interested in studying mean change as measured by the reference method (e.g., self-reports) should at least establish strong MI as proposed above (see Equations 34–35). LS-COM models implying different degrees of MI can be compared by using a χ^2^-difference test. For calculating level-specific χ^2^-difference tests see Ryu and West (2009).

## 9. Simulation study

To investigate the performance of the LS-COM model proposed throughout the previous sections, a Monte Carlo (MC) simulation study was performed. The main purpose of the simulation was to examine the applicability of the LS-COM model across a range of conditions and to establish a set of guidelines and recommendations concerning sample size and model complexity that ensure consistent and unbiased estimation of parameters and their standard errors and minimize potential estimation problems (so called Heywood cases).

### 9.1. Results of previous simulation studies

Numerous simulation studies have been performed in the past focusing on the applicability and robustness of the single-level (classical) SEMs (e.g., Boomsma, 1982; Gerbing and Anderson, 1985; MacKinnon et al., 1995; Marsh et al., 1998; Fan et al., 1999; Raykov, 2000; Enders and Bandalos, 2001; Jackson, 2001; Bandalos, 2002). So far, only few simulation studies have been carried out investigating complex multilevel structural equation models (e.g., Satorra and Muthen, 1995; Hox and Maas, 2001; Julian, 2001; Stapleton, 2002; Maas and Hox, 2005) or longitudinal CFA-MTMM models (Crayen, 2008; Geiser, 2009). In this section, we briefly summarize the results of previous simulation studies that are most relevant to the present study.

With regard to single-level (classical) SEMs a ratio of 5 (sometimes 10) observations per parameter has been suggested to ensure reliable parameter estimates and standard errors (Bentler and Chou, 1987; Bollen, 1989, 2002). With regard to multilevel (two level) SEMs, simulation studies indicate that the number of Level 2 units are more important than the number of Level 1 units suggesting that at least 100 Level 2 units be sampled for accurate standard error estimates and for detecting small effects on Level 2 (Hox and Maas, 2001; Maas and Hox, 2005; Meuleman and Billiet, 2009). It has also been found that ignoring the multilevel structure completely can lead to biased parameter estimates as well as their standard errors (Julian, 2001). Recent simulation studies favor the use of Bayesian estimation techniques showing that 20 Level 2 units can be sufficient for reliable parameter estimates when using weakly informative priors (Hox et al., 2012). Nevertheless for maximum likelihood estimation, it has been generally recommended to sample at least 100 Level 2 units to ensure reliable parameter and standard error estimates (Hox and Maas, 2001; Maas and Hox, 2005; Meuleman and Billiet, 2009).

Simulation studies examining the statistical performance of longitudinal CFA-MTMM [i.e., CS-C(M-1)] models have shown that the parameter estimates and their standard errors are well-recovered in general. Nevertheless, the standard errors seem to be more sensitive to bias than the parameter estimates (Crayen, 2008; Geiser, 2009). Moreover, the statistical performance of the CS-C(M-1) model increases with larger sample sizes (i.e., more empirical informations), fewer constructs and methods (i.e., less complex models) and with low convergent validity (i.e., increasing method bias) (Crayen, 2008; Geiser, 2009).

Given that the CS-C(M-1) model by Geiser (2009) is a single-level confirmatory factor model, it is not clear to which extent the results described above apply to the LS-COM model. Similarly, the results of the simulation studies examining the performance of multilevel structural equation models (ML-SEM) may also not apply to the LS-COM model, given that the models used in those simulation studies are usually less complex (including only a few latent factors and no complex MTMM structure).

### 9.2. Design of the simulation study

To investigate the effect of model complexity and sample size on estimation problems and precision it was necessary to vary a number of potentially influential factors. Because the LS-COM model is a longitudinal multilevel CFA-MTMM model, three main factors influence model complexity. To allow distinguishing their influences (a) the number of constructs (1 vs. 2), (b) the number of methods (2 vs. 3), and (c) the number of occasions of measurement (2, 3, and 4) were varied independently.

In addition to these sources of model complexity, real-life applications of MTMM analysis vary greatly in the degree of convergent validity between the employed methods. To investigate whether convergent validity has an effect on the quality of the estimation this factor was also varied in two levels (high vs. low convergent validity). We used the coefficients of consistency, method specificity, and reliability to specify the true (population) model parameters. The degree of consistency and method specificity were allowed to differ across MC conditions, implying a condition of high consistency (i.e., high convergent validity) and a condition of low consistency (i.e., low convergent validity). The reliability of each indicator was obtained by the sum of the consistency and method specificity coefficients (range: 0.775–0.825). Table 2 shows the population values for the different variance components for the different indicators.

**Table 2:** **Consistency, method specificity and reliability of the LS-COM model**.

	**Low consistency**		**High consistency**	
	**Mean**	**SD**	**Mean**	**SD**
Consistency	0.30	(± 0.025)	0.60	(± 0.025)
Unique method specificity	0.25	(± 0.025)	0.10	(± 0.025)
Common method specificity	0.25	(± 0.025)	0.10	(± 0.025)
Method specificity	0.50	(± 0.050)	0.20	(± 0.050)
Reliability	0.80	(± 0.025)	0.80	(± 0.025)

*The variance coefficients above were standardized with regard to the observed variance of an indicator. Values in parentheses indicate the variation in standard deviations of the consistency and method specificity values across different indicators*.

Due to the multilevel structure of the LS-COM model sample size can be varied on the level of targets (Level 2) as well as on the level of the interchangeable raters (Level 1). As with model complexity, these two factors were varied independently of each other. The number of Level 2 units was set at 100, 250, and 500 targets (Level 2), while the number Level 1 units was set at 2, 5, 10, and 20 raters per target.

In total this simulation design resulted in 2 × 2 × 2 × 3 × 4 × 3 = 288 possible conditions. Of these 288 only 232 were included, because the remaining 56 conditions represented constellations in which the model is underidentified due to there being fewer targets than free model parameters. Of these 56 conditions 50 were conditions with only 100 Level 2 units and all but 8 were conditions represented models with 2 constructs.

Overall, 116,000 (232 × 500) data sets with a varying number or observations (200–10,000) were simulated using Mplus 6.1 (Muthén and Muthén, 2010), the free software R 2.14.0 (R Core Team, 2014), as well as various R packages such as MplusAutomation (Hallquist, 2011), OpenMx (Boker et al., 2011), and corcounts (Erhardt, 2013). All files of this simulation study can be downloaded from the following website^3^. An example Mplus syntax for the simulations study is provided in Appendix B in Supplementary Material.

Strong MI was assumed in all models (c.f. Widaman and Reise, 1997). All models were estimated using the maximum likelihood estimator implemented in M*plus* assuming multivariate normally distributed and complete data.

### 9.3. Evaluation criteria

The performance of the LS-COM model was examined using the following criteria: (a) rate of non-convergence after a maximum of 1000 iterations, (b) rate of improper solutions^4^ (i.e., Heywood cases) due to non-positive definite covariance matrices Ψ and Θ, (c) the amount of parameter estimation as well as standard error bias, and (d) the accuracy of the χ^2^-model fit statistics.

The absolute parameter bias was first calculated for each parameter *p* and then aggregated across all parameters of the same parameter type *c* for which effects were presumed to be equal (e.g., all common method factor loadings, λ_*CMij*3*l*_; c.f. Bandalos, 2006):

(36) $$peb(c)=\frac{1}{n_{c}}\sum_{c=1}^{n_{c}}\left(\frac{\left|M_{pc}-e_{pc}\right|}{e_{pc}}\right).$$

*M*_*pc*_ is the average of the MC parameter estimates across all 500 replications for parameter *p* of parameter type *c*, whereas *e*_*pc*_ is the true population value of that parameter. *n*_*c*_ is the number of parameters in cluster *c*.

In a similar way, the absolute standard error bias was calculated:

(37) $$seb(c)=\frac{1}{n_{c}}\sum_{c=1}^{n_{c}}\left(\frac{\left|M_{SE_{pc}}-SD_{pc}\right|}{SD_{pc}}\right).$$

*M*_*SE*_*pc*__ is the average standard error of parameter *p* allotted to parameter type *c* across all 500 MC replications, whereas *SD*_*pc*_ is the standard deviation of the parameter estimate for parameter *p* in cluster *c* across all 500 MC replications.

The aggregation of the absolute parameter estimation and standard error biases was done for two reasons. First, the LS-COM model incorporates many free parameters to be estimated (sometimes more than 100) and it would not be feasible to report bias for each single model parameter. Second, it is reasonable to assume that similar parameters (e.g., all measurement error variances) are biased in a similar way. Hence, by aggregating parameters that belong to the same parameter type, it was possible to investigate general bias in parameter estimates and their standard errors. In total 12 types of parameters were defined. Eight of these stemmed from the between part of the model: (1) the state factor loadings (λ_*S*_), (2) the common method factor loadings (λ_*CM*_), (3) the method factor loadings (λ_*M*_), (4) the covariances of latent variables on Level 2 (*cov*_*L*2_), (5) the latent means (μ), (6) the latent intercepts (α), (7) the variance of the latent variables at Level 2 (*var*_*L*2_), and (8) the Level 2 residual variances (ϵ_*L*2_). The remaining four parameter clusters all pertained to parameters at Level 1: (9) the unique method factor loadings (λ_*UM*_), (10) the unique method factor variances (*var*_*L*1_), (11) the covariances of the unique method factors (*cov*_*L*1_), and (12) the Level 1 residual variances (ϵ_*L*1_).

In line with previous MC simulation studies investigating MTMM-SEMs (e.g., Nussbeck et al., 2006; Geiser, 2009) 0.10 was chosen as a cut-off criterion for both parameter and SE biases, and absolute values beyond this threshold were deemed unacceptable.

## 10. Results

### 10.1. Rate of non-convergence

All 116,000 specified LS-COM (H0) models converged properly within 1000 iterations.

### 10.2. Rate of improper solutions

M*plus* warning messages regarding potential Ψ-problems were encountered in 65 out of 232 (28.02%) MC conditions, but in only 2,366 out of 116,000 (2.04%) total replications in the simulation. The main reason for the Ψ-warning messages were linear dependencies in the latent covariance matrix due to higher order partial correlations above |1|. Moreover, only 2 out of 232 MC conditions contained improper solutions with regard to latent residual matrix Θ. Hence, the actual amount of improper solutions with regard to this simulation study was below 5%.

**Figure 5:**
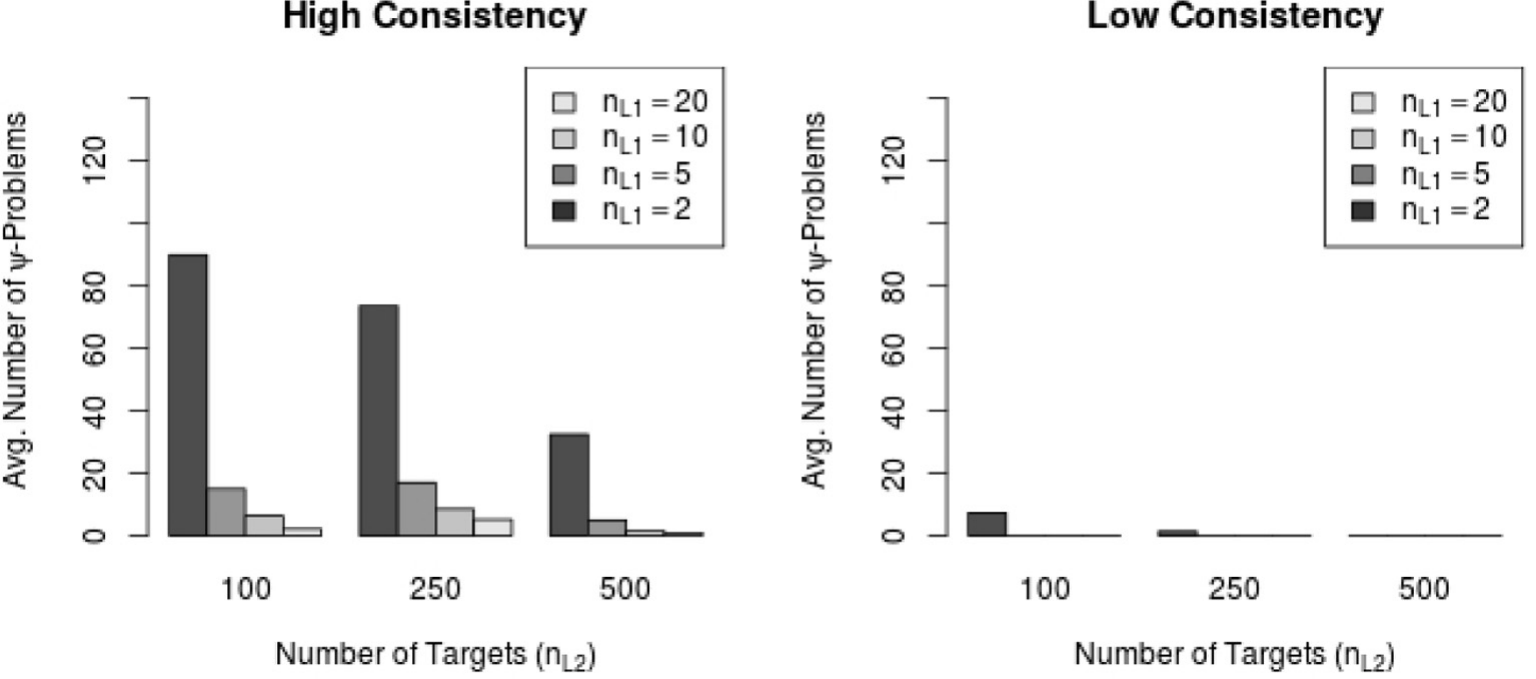
**Average number of Ψ-warning messages in high and low consistency conditions**. *n*_*L*1_ = number of Level 1 units; *n*_*L*2_ = number of Level 2 units.

Most of the conditions exhibiting general warning messages were high consistency conditions (i.e., 56 MC conditions and 2,306 out of 116,000 replications, 1.99%) and only few were low consistency conditions (i.e., 9 MC conditions and 60 out of 116,000 replications, 0.05%) Moreover, the frequency of Ψ-warning messages decreased with increasing sample size on Level 1 (number of raters per target) as well as with increasing sample size on Level 2 (number of targets). Figure 5 shows the relationship between the average amount of Ψ-warning messages and the sample size on both levels in the low and the high consistency conditions. Figure 5 shows that the amount of Ψ-warning messages decreased substantially with the number of targets as well as with the number of raters per target. Figure 5 also indicates that the number of raters per target might be more important for the reduction of Ψ-warning messages than the number of Level 2 units (here: targets).

### 10.3. Amount of parameter and standard error bias

**Figure 6:**
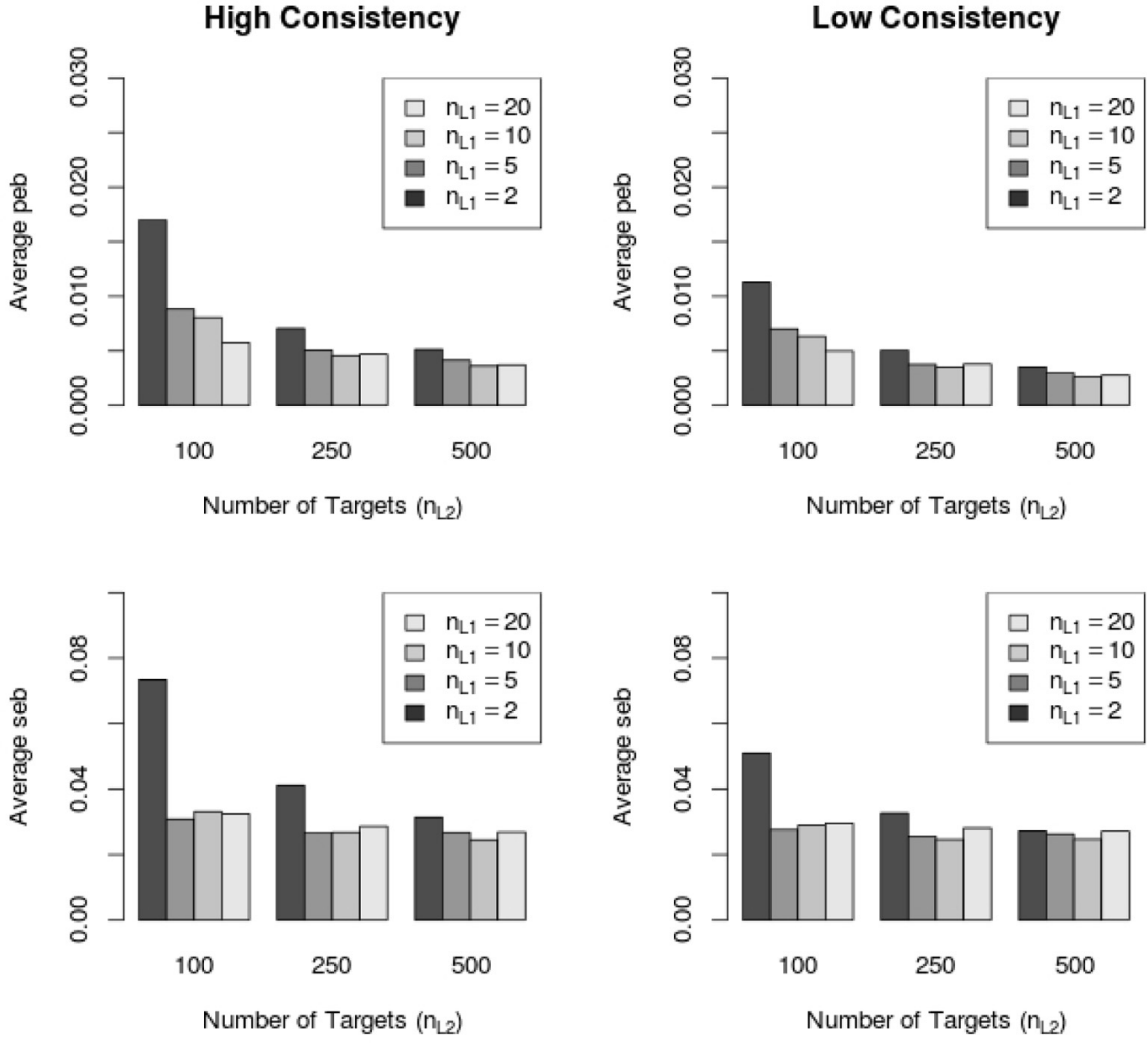
**Average peb and seb values with respect to sample size in high and low consistency conditions in the LS-COM model**. *n*_*L*1_ = number of Level 1 units; *n*_*L*2_ = number of Level 2 units.

Across all 232 conditions the absolute parameter estimation bias (peb, see Equation 36) was below the cutoff value of 10%. However, the absolute standard error bias (seb, see Equation 37) exceeded the value of 10% in 21 out of 232 MC conditions. Higher seb values were more often found in the high consistency (14 out of 21 conditions, 66.67%) conditions than in the low consistency (7 out of 21 conditions, 33.33%) conditions. Figure 6 shows the average peb and seb values across all parameters with respect to the sample size on Level 1 and Level 2 as well as with respect to the consistency condition (high vs low).

**Figure 7:**
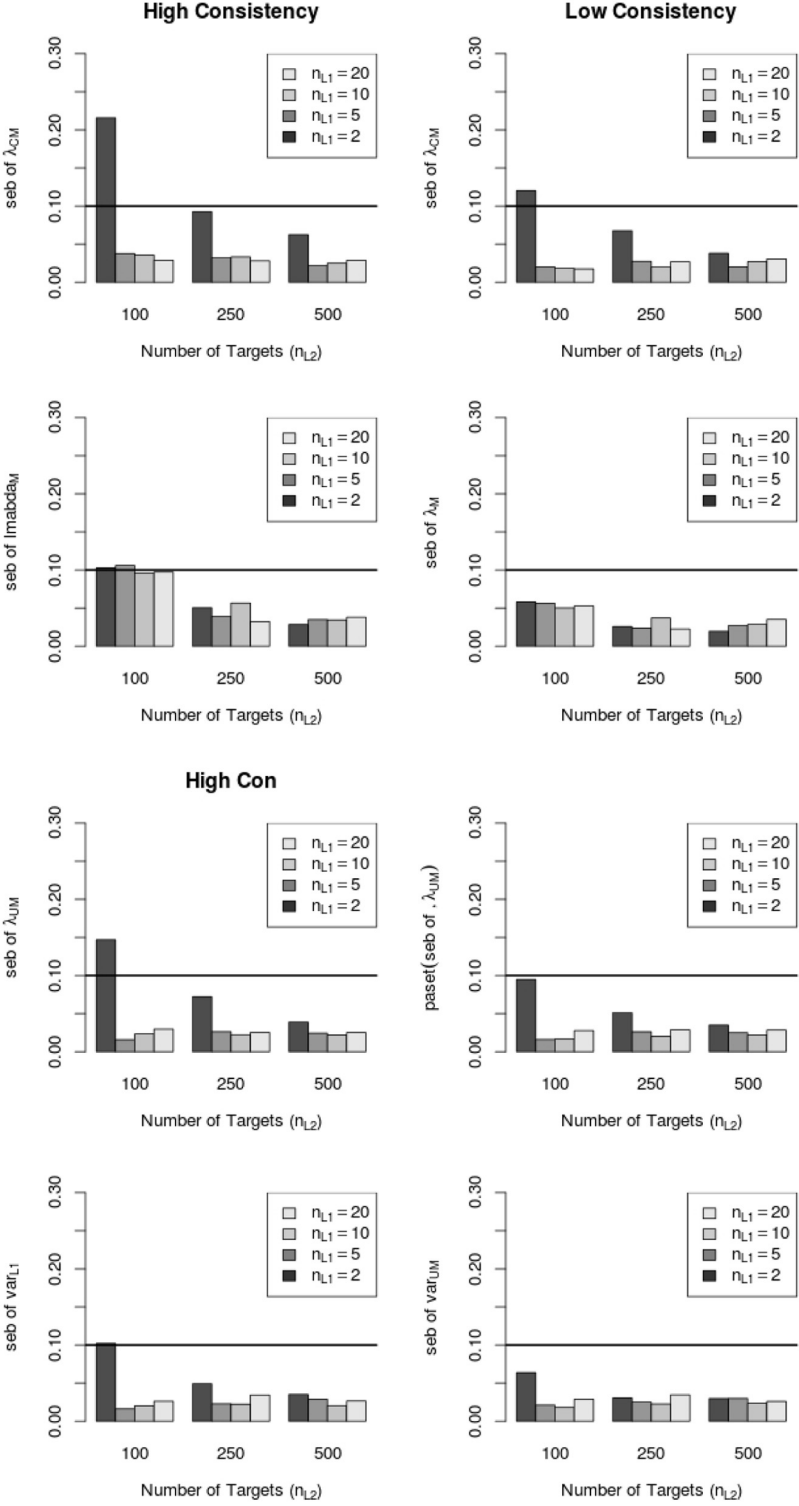
**Relationship between average standard error bias (seb) and sample size for different LS-COM model parameters in the high and low consistency condition**. λ_*CM*_ = common method factor loading parameters; λ_*M*_ = method factor loading parameters; λ_*UM*_ = unique method factor loading parameters; *var*_*L*1_ variance of the unique method factors.

Figure 6 shows that the average peb and seb values decreased with increasing sample size on Level 1 and Level 2. In particular, the sample size on Level 1 (number of raters per target) seemed to be crucial for the reduction of the seb. Moreover, Figure 6 shows that the average amount of peb and seb was lower in the low consistency condition than in the high consistency condition. Note that the average peb and seb (i.e., across all parameters) were below 10% (see Figure 6). Further investigations revealed that specific LS-COM model parameters were more sensitive to bias than others. Specifically, the common method factor loadings λ_*CM*_, method factor loadings λ_*M*_, unique method factor loadings λ_*UM*_, as well as the variances of unique method factors *var*_*L*1_ showed the largest standard error biases. Additionally, the seb of the latent means on Level 2 exceeded the cutoff value of 10% in one single MC condition (i.e., one construct, two methods, two occasions of measurement, 10 Level 1 units and 100 Level 2 units). Figure 7 shows the dependency of the seb values on the sample size at each of the measurement levels in the high and low consistency condition.

According to Figure 7, the standard error bias was substantially reduced with increasing sample size on both levels. In particular, the standard error bias dropped below the cutoff value of 10% when more than 2 raters per target were sampled.

### 10.4. χ^2^-fit-statistics

**Figure 8:**
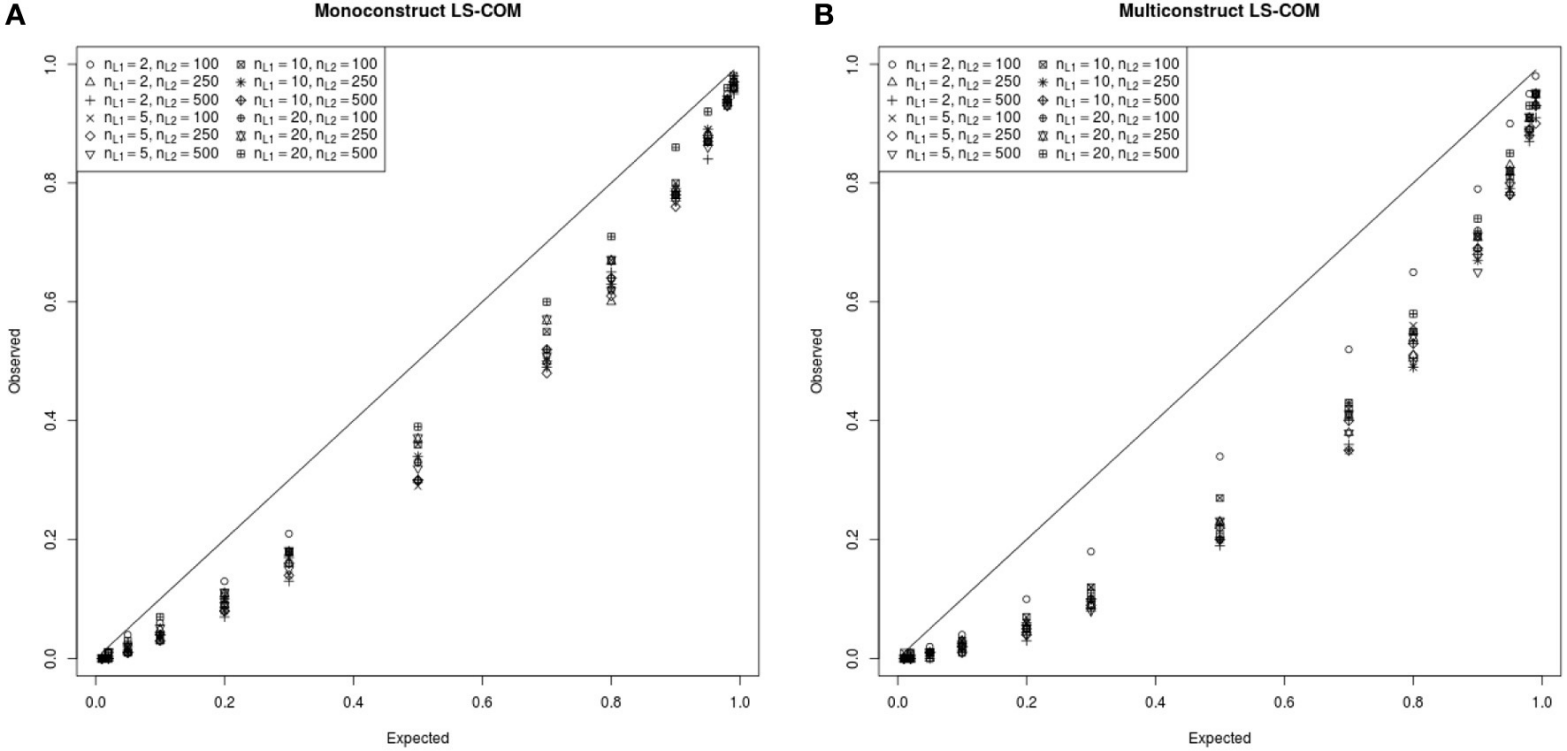
**PP-plot of the observed and theoretical proportions of the χ^2^ values for the monoconstruct LS-COM model (A) and multiconstruct LS-COM model (B) for different sample sizes**.

In Figure 8A,B the simulated and expected proportions of the χ^2^ values for monoconstruct and multiconstruct LS-COM models are presented. According to these results, the simulated χ^2^-values were always below the theoretically expected χ^2^-values indicating a downward bias in the asymptotic type I error. These results suggest that too many specified LS-COM models would be accepted with respect to a nominal alpha level of 0.05 if researchers used the theoretical χ^2^ distribution to test the model fit. Hence, the χ^2^ model fit test appeared to be too liberal with respect to LS-COM models under the conditions studied here. However, the differences between the observed and the expected χ^2^-distributions at a nominal alpha level of 5% were relatively small (on average 0.03 for monoconstruct condition and 0.04 for the multiconstruct condition). The results also indicate that the χ^2^ model fit test was more accurate for less complex (i.e., monoconstruct) LS-COM models. We did not find a straightforward relationship between sample size and the accuracy of the χ^2^ model fit test for the LS-COM model.

## 11. Discussion

In the present work a multilevel longitudinal CFA-MTMM model for the combination of structurally different and interchangeable methods (called LS-COM model) was proposed. The LS-COM model combines the advantages of multilevel, longitudinal, and CFA-MTMM modeling approaches and is suitable for MTMM measurement designs combining different types of methods. Given that such complex MTMM measurement designs are increasingly used in psychology (e.g., 360° feedback designs, multisource, mutirater designs), the LS-COM fills a gap in the current literature on longitudinal MTMM modeling. Previous studies on longitudinal MTMM modeling have either focused exclusively on single-indicator models or on a specific type of method (e.g., structurally different methods) (e.g., Kenny and Zautra, 2001; Burns and Haynes, 2006; Courvoisier et al., 2008; Grimm et al., 2009; Geiser et al., 2010). In the present article a new CFA-MTMM model has been developed allowing the simultaneous analysis of different types of methods (i.e., structurally different and interchangeable methods) across time using a multiple indicator, multilevel latent variable approach. The LS-COM model overcomes many limitations of previous models by allowing researchers to

1. study method effects on different levels (rater and target level),
2. analyze the stability and change of construct and method effects across time,
3. evaluate the convergent and discriminant validity among different methods across time,
4. investigate the stability and change of a given construct (attribute) across time,
5. examine different variance coefficients and the psychometric properties of the measures on multiple occasions of measurement,
6. test important assumptions (e.g., measurement invariance), and
7. study potential causes of method effects by including external variables in the model.

Moreover, the LS-COM model is defined based on the stochastic measurement theory (Suppes and Zinnes, 1963; Zimmerman, 1975; Steyer and Eid, 2001), which bears the advantage of defining the latent variables as random variables with a clear psychometric interpretation. That means that the latent variables in the LS-COM model are not simply assumed, but properly defined as random variables on the probability space (see Appendix A in Supplementary Material for the formal definitions). In addition, the LS-COM makes use of a latent regression modeling [CT-C(M-1)] approach, which allows contrasting different methods against a reference method. The CT-C(M-1) modeling approach bears the advantages of using “pure” method factors by defining the method variables as latent residual variables (see Geiser et al., 2008, for more details). In addition, the CT-C(M-1) modeling approach allows separating the total variance of each indicator into state, method, and measurement error components and calculating different variance coefficients (e.g., coefficients of consistency, method specificity, reliability), which is not possible in other MTMM modeling approaches [e.g., latent means (Pohl and Steyer, 2010) and latent difference modeling (Pohl et al., 2008) approaches].

Researchers who are interested in studying the mean change of an attribute across time should first test the degree of measurement invariance and then estimate the latent means of the latent state factors as described above. In addition, the stability and change of the interindividual differences in an attribute can be investigated by the correlations of the latent state factors pertaining to different occasions of measurement. The stability and change of the method factors across time can be studied with regard to the correlations between the latent method factors measured on different occasions of measurement. In total, three different types of method effects can be examined. First, the method effects of the structurally different method (e.g., parent reports) that is not shared with the reference method (e.g., self-reports). Second, the common method effect of the interchangeable methods (e.g., general peer rating) that is not shared with the reference method (e.g., self-report). Third, the unique method effect of the interchangeable methods (e.g., single peer rating) that is neither shared with the reference method (e.g., self-reports), nor with other peers. A meaningful interpretation of correlation coefficients between method factors across time (e.g., as stability of method effects), typically requires that the same raters are recruited at each time point. The generalizability of the method effects can be examined by the correlations of latent method factors pertaining to different types of methods (structurally different and interchangeable methods).

In order to examine the trustworthiness of the parameter and standard error estimates in the LS-COM model, we conducted a MC simulation study. To our knowledge, no simulation study has been performed so far scrutinizing the statistical performance of complex longitudinal, multilevel, multiple indicator CFA-MTMM models.

According to the results of our MC simulation study, the LS-COM model can produce reliable parameter estimates even in small samples with just 100 targets and 2 raters per targets. However, for such small samples the standard errors of LS-COM model parameters will be marginally biased. Most sensitive to bias are the standard errors of the method factor loading parameters (i.e., λ_*UMijkl*_, λ_*CMijkl*_, λ_*Mijkl*_) as well as the standard errors of the unique method factor variance [i.e., *V*ar(*UM*_*rtjkl*_)]. The standard error bias can be reduced by increasing the number of Level 1 units (i.e., number of raters per target). In cases with at least 5 raters per target and 100 targets, the LS-COM produced unbiased parameters as well as standard errors in our simulation. In general, parameter estimates seemed more accurate in cases with low convergent validity. Low convergent validity is often seen in practice (e.g., Eid et al., 2003, 2008; Carretero-Dios et al., 2011; Pham et al., 2012), so that the LS-COM model should generally result in unbiased parameter and SE estimates.

The number of methods as well as the number of occasions of measurement did not seem to affect the accuracy of the parameter estimation or their standard errors. If at all, more occasions of measurement proved beneficial for the stability of the parameter estimation. This is most likely due to the fact that strong MI was assumed for the repeated measures in the simulation. Because of this, the ratio of available information to free parameters actually increased with more measurement occasions. It should be noted, however, that this condition might not be present in applications in which the assumption of strong MI does not hold or the number of occasions is very large.

In contrast to the number measurement occasions an increasing number of constructs generally does make the LS-COM model more complex, because invariance assumptions are generally not imposed across different constructs. In cases with many constructs, we recommend splitting the complete LS-COM model into multiple submodels and analyzing all combinations using two constructs simultaneously. All coefficients of interest (e.g., correlations) can still be estimated without affecting the meaning of any parameter in the model. A prerequisite for the step-by-step procedure is that the same reference method is chosen.

The results of this simulation study support previous findings of classical SEM (see Bentler and Chou, 1987; Bollen, 1989, 2002). Based on a simulation study, Bentler and Chou (1987) suggested that a ratio of 5:1 (observations per parameter) is sufficient for proper parameter estimates with regard to classical structural equation models. The results of our simulation study support this conclusion for LS-COM models. We therefore recommend sampling at least 5 raters per target and at least as many targets as there are free parameters to be estimated. Our simulation study also revealed new insights into complex multilevel SEM, namely that the sample size on Level 1 is an important factor that influences the quality of model estimation. Previous simulation studies devoted to this research area claimed that the number of Level 1 units is less important than the number of Level 2 units (Maas and Hox, 2005). Our results show that the number of Level 1 units can be crucial for the reduction of standard error bias in complex multilevel structural equation models.

So far, only few studies have investigated the accuracy of χ^2^-fit-statistics in complex ML-SEMs (Ryu and West, 2009; Ryu, 2014; Schermelleh-Engel et al., 2014). The results of our simulation study are generally encouraging as they indicated that the overall χ^2^-test of exact fit was only marginally biased with regard to a nominal alpha level of 5% and multivariate normal distributed and complete data. More specifically, our results indicate that the overall maximum likelihood χ^2^-test of exact fit may be slightly too liberal for complex ML-SEM models. However, we recommended to use robust maximum likelihood estimation (MLR) when multivariate normality cannot be assumed.

Future studies should focus on three issues associated with complex longitudinal multilevel MTMM modeling. First, the statistical effects of attrition (i.e., missingness) of the interchangeable raters across time and the possibilities of alternative modeling approaches should be investigated. Second, the robustness of χ^2^ fit statistics in complex multilevel SEM with non-normal and (un)complete data should be examined and alternative fit statistics for complex multilevel SEMs should be scrutinized. With respect to the investigation of fit statistics in multilevel SEM, researchers maybe inspired by the recent work of Schermelleh-Engel et al. (2014) and Ryu (2014). Third, future studies should focus on possible extensions of the LS-COM model to the other longitudinal modeling approaches [e.g., latent state-trait models, latent difference (change) models, latent growth curve models] with one or more sets of interchangeable methods and apply these models to real data.

## 12. Conclusion and general recommendation

In this work, we presented a new longitudinal multilevel CFA-MTMM model for the combination of structurally different and interchangeable methods. The model extends the spectrum of longitudinal MTMM modeling approaches by allowing the simultaneous investigation of method effects on different measurement levels across time. With respect to the results of the simulation study, we recommend that researchers should sample at least as many Level 2 units (i.e., targets) as there are free parameters to be estimated in the model and at least 5 interchangeable raters per target in order to obtain a reliable sample size for proper parameter standard error estimation. Moreover, we suggest that researchers should test the degree of MI when studying mean change of a given attribute across time.

## Author note

Dr. Tobias Koch: Note that the psychometric definition of the LS-COM model and the simulation study were previously presented as part of the doctoral thesis by Koch (2013). This research was funded by the German Research Foundation (Deutsche Forschungsgesellschaft, DFG, grant number: EI 379/6-1). Christian Geiser′s work was funded by a grant from the National Institutes on Drug Abuse (NIH-NIDA), grant number: 1 R01 DA034770-01. The content is solely the responsibility of the authors and does not necessarily represent the official views of the NIH.

### Conflict of interest statement

The authors declare that the research was conducted in the absence of any commercial or financial relationships that could be construed as a potential conflict of interest.

## Footnotes

^1^For more details on partial (MI) see Byrne et al. (1989) and on approximate measurement invariance see Van De Schoot et al. (2013).

^2^Another possibility is to set the intercept of a reference indicator (e.g., first indicator) to zero on all occasions of measurement and estimate the latent means of the latent state factor on each occasion of measurement.

^3^http://www.ewi-psy.fu-berlin.de/einrichtungen/arbeitsbereiche/psymeth/mitarbeiter/tkoch/index.html.

^4^Ψ-warning messages indicate linear dependencies in the covariance matrix of the latent variables, whereas Θ-warning messages indicate estimation problems with regard to the latent error variables (e.g., negative error variances).
