# Supplementary material for: A longitudinal multilevel CFA-MTMM model for interchangeable and structurally different methods
Source: Front Psychol. 2014 Apr 17;5:311. doi: 10.3389/fpsyg.2014.00311 (PMC4029009; doi:10.3389/fpsyg.2014.00311)
Supplement: Supplementary file 1 [file Presentation1.PDF]

Appendix A

Definition 1 (LS-COM model.) *The random variables* $\{Y_{111111}, \ldots, Y_{rtijkl}, \ldots, Y_{abcdef}\}$ *and* $\{Y_{11111}, \ldots, Y_{tijkl}, \ldots, Y_{bcdef}\}$ *on a probability space* $(\Omega, \mathfrak{A}, P)$ *are variables of a LS-COM model if the following conditions hold:*

(a) $(\Omega, \mathfrak{A}, P)$ *is a probability space such that* $\Omega = \Omega_T \times \Omega_{TS_1} \times \ldots \times \Omega_{TS_l} \times \Omega_R \times \Omega_{RS_1} \times \ldots \times \Omega_{RS_l} \times \Omega_{ijk1} \times \ldots \times \Omega_{ijkl}$, *where* Ω *is the set of all possible samples,* Ω_T *is the set of possible targets,* Ω_{TS_l} *is the set of possible target-situations,* Ω_R *is the set of possible raters,* Ω_{RS_l} *is the set of possible rater-situations, and* Ω_{ijkl} *is the set of possible outcomes with respect to indicator* i*, construct* j*, method* k*, and occasions of measurement* l*.*

(b) The projections $p_T : \Omega \to \Omega_T$, $p_{TS_l} : \Omega \to \Omega_{TS_l}$, $p_R : \Omega \to \Omega_R$, *and* $p_{RS_l} : \Omega \to \Omega_{RS_l}$ *are random variables on* $(\Omega, \mathfrak{A}, P)$*.*

(c) The Level 2 observed variables $Y_{tijkl} : \Omega_T \times \Omega_{TS_1} \times \ldots \times \Omega_{TS_l} \to \mathbb{R}$ *and the Level 1 observed variables* $Y_{rtijkl} : \Omega_T \times \Omega_{TS_1} \times \ldots \times \Omega_{TS_l} \times \Omega_R \times \Omega_{RS_1} \times \ldots \times \Omega_{RS_l} \to \mathbb{R}$, *for which* $r \in R \equiv \{1, \ldots, a\}$, $t \in T \equiv \{1, \ldots, b\}$, $i \in I \equiv \{1, \ldots, c\}$, $j \in J \equiv \{1, \ldots, d\}$, $k \in K \equiv \{1, \ldots, e\}$, $l \in L \equiv \{1, \ldots, f\}$ *are random variables on* $(\Omega, \mathfrak{A}, P)$ *with finite first- and second-order moments.*

(d) Without loss of generality, the first method (k = 1) is selected as reference method. The second method (k = 2) refers to another structurally different method, which serves as nonreference method, and (k=3) is the set of interchangeable methods, which also serves as nonreference method. Then, the following (Level 1 and Level 2) latent variables are random variables on $(\Omega, \mathfrak{A}, P)$ *with finite first- and second-order moments:*

Level 1 (the rater level):

$$S_{rtij3l} \equiv E(Y_{rtij3l} | p_T, p_{TS_l}, p_R, p_{RS_l}), \tag{38}$$
$$UM_{rtij3l} \equiv S_{rtij3l} - E(S_{rtij3l} | p_T, p_{TS_l}), \tag{39}$$
$$\epsilon_{rtij3l} \equiv Y_{rtij3l} - E(Y_{rtij3l} | p_T, p_{TS_l}, p_R, p_{RS_l}). \tag{40}$$

Level 2 (the target level):

$$S_{tijkl} \equiv E(Y_{tijkl} | p_T, p_{TS_l}), \tag{41}$$
$$S_{tij3l} \equiv E(S_{rtij3l} | p_T, p_{TS_l}), \tag{42}$$
$$M_{tij2l} \equiv S_{tij2l} - E(S_{tij2l} | S_{tij1l}), \tag{43}$$
$$CM_{tij3l} \equiv S_{tij3l} - E(S_{tij3l} | S_{tij1l}), \tag{44}$$
$$\epsilon_{tijkl} \equiv Y_{tijkl} - E(Y_{tijkl} | p_T, p_{TS_l}). \tag{45}$$

(e) For each construct j*, measured by a nonreference method* ($k \neq 1$) *on occasion of measurement* l *with item* i*, there is a constant* $\alpha_{ijkl} \in \mathbb{R}$ *as well as a constant*

$\lambda_{Sijkl} \in \mathbb{R}_+$ *such that*

$$E(S_{tijkl}|S_{tij1l}) = \alpha_{ijkl} + \lambda_{Sijkl} S_{tij1l}. \tag{46}$$

(f) For each construct j, measured by nonreference method 2 (the structurally different method) on occasion of measurement l and for each pair $(i, i') \in I \times I'$, $(i \neq i')$ *there is a constant* $\lambda_{Mii'j2l} \in \mathbb{R}_+$ *such that*

$$M_{tij2l} = \lambda_{Mii'j2l} M_{ti'j2l}. \tag{47}$$

(g) For each construct j, measured by nonreference method 3 (the set of interchangeable methods) on occasion of measurement l and for each pair (i, i') $\in I \times I'$, $(i \neq i')$ *there is a constant* $\lambda_{UMii'j3l} \in \mathbb{R}_+$ *such that*

$$UM_{rtij3l} = \lambda_{UMii'j3l} UM_{rti'j3l}. \tag{48}$$

(h) For each construct j, measured by nonreference method 3 on occasion of measurement l and for each pair $(i, i') \in I \times I'$, $(i \neq i')$ *there is a constant* $\lambda_{CMii'j3l} \in \mathbb{R}_+$ *such that*

$$CM_{tij3l} = \lambda_{CMii'j3l} CM_{ti'j3l}. \tag{49}$$

Appendix B

```
Title: LS-COM Simulation Template
       Low Consistency
       2 Constructs
       2 Methods
       2 Occasions
Montecarlo: ! Set to monte carlo  for simulation study
   names=Y1111 Y2111 Y3111 ! observed variables
               Y1121 Y2121 Y3121 ! indicators i=3
               Y1211 Y2211 Y3211 ! constructs j=2
               Y1221 Y2221 Y3221 ! methods k=2
               Y1112 Y2112 Y3112 ! occasions l=2
               Y1122 Y2122 Y3122 ! Yijkl
               Y1212 Y2212 Y3212
               Y1222 Y2222 Y3222;
   nreps=500; ! number of replications
   nobservations=500; ! number of total observations
   Ncsizes=1;
```

```
    Csizes=100(5); ! 100 targets(5 rater per target)
    seed=55719;
    !Repsave=All; ! saves all simulated data files
    !Save=Data*.dat; ! saves all simulated data files
Between=Y1111 Y2111 Y3111 ! observed variables on level two
              Y1211 Y2211 Y3211 ! exist only for the fixed methods
              Y1112 Y2112 Y3112 ! not for the random methods!!!
              Y1212 Y2212 Y3212;
Model Population:
%Within%
!!!!UNIQUE METHOD FACTORS!!!!
!Factor Loadings
UM121 by Y1121@1          ! first factor loading is set to 1 for identification
         Y2121*0.953462589 (LUM212) ! set factor loadings equal across time
         Y3121*0.90453394 (LUM312);
UM221 by Y1221@1!
         Y2221*0.953462589 (LUM222)
         Y3221*0.90453394 (LUM322);
UM122 by Y1122@1
         Y2122*0.953462589 (LUM212)
         Y3122*0.90453394 (LUM312);
UM222 by Y1222@1
         Y2222*0.953462589 (LUM222)
         Y3222*0.90453394 (LUM322);
!Factor Variance
UM121*0.275; ! freely estimated
UM221*0.275;
UM122*0.275;
UM222*0.275;
!Factor Mean Structure (is default in Mplus)
[UM121@0]; ! fix all means of the UM-factor to zero
[UM221@0];
[UM122@0];
[UM222@0];
!!!!RESIDUAL VARIANCES!!!!
```

```
Y1121*0.175; ! freely estimated
Y2121*0.2;
Y3121*0.225;
Y1221*0.175;
Y2221*0.2;
Y3221*0.225;
Y1122*0.175;
Y2122*0.2;
Y3122*0.225;
Y1222*0.175;
Y2222*0.2;
Y3222*0.225;
!!!!COVARIANCES!!!!
UM121 with UM221*0.0825
           UM122*0.165
           UM222*0.0275;
UM221 with UM122*0.0275
           UM222*0.165;
UM122 with UM222*0.0825;
%BETWEEN%
!!!!STATE FACTORS!!!!
!Factor Loadings
S111 by Y1111@1   ! first factor loading is set to 1 for identification
        Y2111*0.984731938 (LS211) ! set factor loadings equal across time
        Y3111*0.969223322 (LS311)
        Y1121*0.627645901 (LS112)
        Y2121*0.603022736 (LS212)
        Y3121*0.577350243 (LS312);
S211 by Y1211@1
        Y2211*0.984731938 (LS221)
        Y3211*0.969223322 (LS321)
        Y1221*0.627645901 (LS122)
        Y2221*0.603022736 (LS222)
        Y3221*0.577350243 (LS322);
S112 by Y1112@1
```

```
88          Y2112*0.984731938 (LS211)
89          Y3112*0.969223322 (LS311)
90          Y1122*0.627645901 (LS112)
91          Y2122*0.603022736 (LS212)
92          Y3122*0.577350243 (LS312);
93  S212 by Y1212@1
94          Y2212*0.984731938 (LS221)
95          Y3212*0.969223322 (LS321)
96          Y1222*0.627645901 (LS122)
97          Y2222*0.603022736 (LS222)
98          Y3222*0.577350243 (LS322);
99  !Factor Variances
100 S111*0.825; ! freely estimated
101 S211*0.825;
102 S112*0.825;
103 S212*0.825;
104 !Factor Mean Structure
105 [S111@0]; ! set means of the latent state factor
106 [S211@0]; ! on the 1 occasion of measurement to zero
107 [S112*0.3]; ! estimate the means of the latent state factors
108 [S212*0];   ! for the 2 occasion of measurement
109 !!!!COMMON METHOD FACTORS!!!!
110 !Factor Loadings
111 CM121 by Y1121@1     ! first factor loading is set to 1 for identification
112          Y2121*1.054092553 (LM212) ! set factor loadings equal across time
113          Y3121*1.105541546 (LM312);
114 CM221 by Y1221@1
115          Y2221*1.054092553 (LM222)
116          Y3221*1.105541546 (LM322);
117 CM122 by Y1122@1
118          Y2122*1.054092553 (LM212)
119          Y3122*1.105541546 (LM312);
120 CM222 by Y1222@1
121          Y2222*1.054092553 (LM222)
122          Y3222*1.105541546 (LM322);
```

```
123 !Factor Variances
124 CM121*.225; ! freely estimated
125 CM221*.225;
126 CM122*.225;
127 CM222*.225;
128 !Factor Mean Structure
129 [CM121@0]; ! zero means (default in Mplus)
130 [CM221@0];
131 [CM122@0];
132 [CM222@0];
133 !!!!RESIDUAL VARIANCES!!!!
134 Y1111*0.175; ! freely estimated for level 2 indicators
135 Y2111*0.2;
136 Y3111*0.225;
137 Y1121@0; ! fixed to zero for level 1 indicators
138 Y2121@0; ! fixed to zero for level 1 indicators
139 Y3121@0; ! fixed to zero for level 1 indicators
140 Y1211*0.175;
141 Y2211*0.2;
142 Y3211*0.225;
143 Y1221@0; ! fixed to zero for level 1 indicators
144 Y2221@0; ! fixed to zero for level 1 indicators
145 Y3221@0; ! fixed to zero for level 1 indicators
146 Y1112*0.175;
147 Y2112*0.2;
148 Y3112*0.225;
149 Y1122@0; ! fixed to zero for level 1 indicators
150 Y2122@0; ! fixed to zero for level 1 indicators
151 Y3122@0; ! fixed to zero for level 1 indicators
152 Y1212*0.175;
153 Y2212*0.2;
154 Y3212*0.225;
155 Y1222@0; ! fixed to zero for level 1 indicators
156 Y2222@0; ! fixed to zero for level 1 indicators
157 Y3222@0; ! fixed to zero for level 1 indicators
```

```
158 !!!!Intercepts!!!!
159 [Y1111*1] (A111); ! freely estimated (set to 1 here)
160 [Y2111*1] (A211);
161 [Y3111*1] (A311);
162 [Y1121*1] (A112);
163 [Y2121*1] (A212);
164 [Y3121*1] (A312);
165 [Y1211*1] (A121);
166 [Y2211*1] (A221);
167 [Y3211*1] (A321);
168 [Y1221*1] (A122);
169 [Y2221*1] (A222);
170 [Y3221*1] (A322);
171 [Y1112*1] (A111);
172 [Y2112*1] (A211);
173 [Y3112*1] (A311);
174 [Y1122*1] (A112);
175 [Y2122*1] (A212);
176 [Y3122*1] (A312);
177 [Y1212*1] (A121);
178 [Y2212*1] (A221);
179 [Y3212*1] (A321);
180 [Y1222*1] (A122);
181 [Y2222*1] (A222);
182 [Y3222*1] (A322);
183 !!!!COVARIANCES!!!!
184 S111 with CM121@0       ! fix to zero
185           S211*.4125
186           CM221@0               ! fix to zero
187           S112*.495
188           CM122@0              ! fix to zero
189           S212*.2475
190           CM222@0;              ! fix to zero
191 CM121 with S211@0        ! fix to zero
192            CM221*0.0675
```

```
193             S112@0                ! fix to zero
194             CM122*0.135
195             S212@0                ! fix to zero
196             CM222*0.0225;
197 S211 with CM221@0        ! fix to zero
198             S112*0.2475
199             CM122@0               ! fix to zero
200             S212*0.495
201             CM222@0;         ! fix to zero
202 CM221 with S112@0         ! fix to zero
203             CM122*0.0225
204             S212@0                ! fix to zero
205             CM222*0.135;
206 S112 with CM122@0        ! fix to zero
207             S212*0.4125
208             CM222@0;         ! fix to zero
209 CM122 with S212@0         ! fix to zero
210             CM222*0.0675;
211 S212 with CM222@0;        ! fix to zero
212 Analysis:
213     Type=Twolevel; ! for multilevel sem
214     Estimator=ML;  ! or mlr (default in Mplus)
215     Information=Expected;
216     H1Iterations=7500;   ! set H1 iteration to large number
217 Model:
218 repeat input from above
219 Output: Tech9; ! simulation output
```
